# A Confidential Word to the Conscientious X-Ray Man

**Published:** 1917-02

**Authors:** C. N. Johnson


### A CONFIDENTIAL WORD TO THE CONSCIENTIOUS X-RAY MAN.

BY C. N. JOHNSON, A.M., D.D.S.

We need you in our work, and we are very grateful for your services. You have done much good, and are capable of doing much more good. You have also done great harm, and are capable of doing still greater harm. Let us get together and study how this harm may be avoided. In the first place you are assuming altogether too much, and are making many unwarranted statements. The simple fact is that you do not know many of the things you claim to know. You glibly write "pus-pockets," "abscess," "infection," "gross infection," opposite certain teeth when there is positively nothing of the kind associated with those teeth, and what is worse, you hand this false information to the patient, thus frightening him into the removal of useful teeth which can never be successfully replaced. You are too often men with a single purpose—the purpose of finding something. Sometimes you are a dentist earnestly seeking for the truth. Sometimes you are a dentist who has studied dental histology and dental pathology; and who is alert to the problems presented in maintaining the mouth and teeth in a condition of health and usefulness. Sometimes you are a physician who has been well trained in general medicine and in diagnosis but who knows almost nothing about dental histology or pathology, and has not the slightest conception of the significance of saving or losing a tooth. Sometimes you are neither a dentist nor physician and are utterly devoid of knowledge regarding pathological processes. You could not diagnose a mountain torrent from a cataract of the eye, and yet you sagely make a diagnosis of mouth conditions and write it down for the patient to read. In this you may be doing the dental practitioner a grievous wrong—in fact you have done it in many instances. What is infinitely worse, you may be doing the patient irreparable injury—you have done it. You have needlessly frightened many a patient into the extraction of teeth that should never have been removed—leaving the patient in a worse condition than before.

Let us reason together and see if we can not be mutually helpful one to the other, and serve the people better than we are doing. We—the men who do not make X-ray pictures—need your aid in many ways, and we cheerfully accord to you the ability to produce a better picture than we could possibly do. But we want you to remember this one thing and abide by it—you have absolutely no right to diagnose any case and pass your opinion upon it without consultation with the dentist who has had the case in charge. No X-ray evidence should ever be taken except in conjunction with the clinical history of the case. Not that we ask you to merely take a picture and remain passive in the matter. We welcome your opinion stated to us frankly, whether that opinion is right or wrong. You may be able to detect evidence in a picture that we would overlook, and we thank you for calling our attention to it. But we ask you to cease from this time making a diagnosis to the patient, and writing those mystic and formidable legends on X-ray pictures, "pus-pockets," "infection," etc., etc., and handing it to the patient. Why? Not because we want to have the truth hidden, but because we do not want people misled. And in the past people have been woefully misled. You have written "abscess" when there was no abscess. You have diagnosed "pus-pocket" over the apical end of a root, which on investigation contained a perfectly healthy living pulp, and you have condemned teeth on X-ray evidence which were entirely harmless and altogether useful. The simple fact is that you can not tell what is meant by a shadow around the root of a tooth in an X-ray picture, and until you know more about this you have no right to condemn a patient to the agony of apprehension and the terror of uncertainty produced by reading such highly-colored terms.

If you will co-operate with us conscientiously and conservatively in clearing up the many problems which confront us in our work, and in which you can be of such splendid service, we shall take the greatest pleasure in working with you and supporting your efforts; but if you continue in the way some of you are traveling it will not be long before not only the profession but the public will be heaping on your head a righteous malediction, and crying in your ears the unhappy word "Anathema."—Editorial, *Dental Review*.

